# CFTR and Gastrointestinal Cancers: An Update

**DOI:** 10.3390/jpm12060868

**Published:** 2022-05-25

**Authors:** Rahul Bhattacharya, Zachary Blankenheim, Patricia M. Scott, Robert T. Cormier

**Affiliations:** Department of Biomedical Sciences, University of Minnesota Medical School, Duluth, MN 55812, USA or Rahul.Sunnybhattacharya@gmail.com (R.B.); blank054@d.umn.edu (Z.B.)

**Keywords:** cystic fibrosis, CFTR, gastrointestinal cancers, tumor suppressor gene, modulators

## Abstract

Cystic Fibrosis (CF) is a disease caused by mutations in the *CFTR* gene that severely affects the lungs as well as extra-pulmonary tissues, including the gastrointestinal (GI) tract. CFTR dysfunction resulting from either mutations or the downregulation of its expression has been shown to promote carcinogenesis. An example is the enhanced risk for several types of cancer in patients with CF, especially cancers of the GI tract. CFTR also acts as a tumor suppressor in diverse sporadic epithelial cancers in many tissues, primarily due to the silencing of CFTR expression via multiple mechanisms, but especially due to epigenetic regulation. This review provides an update on the latest research linking CFTR-deficiency to GI cancers, in both CF patients and in sporadic GI cancers, with a particular focus on cancer of the intestinal tract. It will discuss changes in the tissue landscape linked to CFTR-deficiency that may promote cancer development such as breakdowns in physical barriers, microbial dysbiosis and inflammation. It will also discuss molecular pathways and mechanisms that act upstream to modulate CFTR expression, such as by epigenetic silencing, as well as molecular pathways that act downstream of CFTR-deficiency, such as the dysregulation of the Wnt/β-catenin and NF-κB signaling pathways. Finally, it will discuss the emerging CFTR modulator drugs that have shown promising results in improving CFTR function in CF patients. The potential impact of these modulator drugs on the treatment and prevention of GI cancers can provide a new example of personalized cancer medicine.

## 1. Introduction

Cystic Fibrosis is a monogenic disease caused by mutations in the *CFTR* gene encoding for the cystic fibrosis transmembrane conductance regulator (CFTR) protein [1]. The CFTR protein is a cAMP-dependent anion transporter that transports chloride (Cl^−^) and bicarbonate (HCO_3_^−^) ions across the apical plasma membrane of epithelial cells. Moreover, this protein regulates the activity of other ion channels, including epithelial sodium ion channels. Therefore, mutations or the absence of CFTR results in an imbalance of ions and fluids in the cells of the airways, intestine, pancreas, and other organs [2]. Mutations in CFTR are generally classified based on the nature of the dysfunction, including aberrant protein production, cellular processing or activity. Mutations in the *CFTR* gene fall into six basic classes. The most common pathogenic mutant form of CFTR is designated as F508del (a phenylalanine residue at site 508 is deleted) and this mutation is most common among Caucasians of northern European descent [3].

With the recent development of improved CF therapies, including CFTR modulator drugs, the life expectancy of patients with CF has increased substantially. However, one major challenge that still remains is the increased predisposition of patients with CF to cancer, as shown by many studies. CFTR has been found to act almost exclusively as a tumor suppressor in different cancer types and dysfunctional CFTR has been associated with the promotion of carcinogenesis [4]. Among the different cancer types, there is an increased risk of patients with CF for gastrointestinal (GI) cancers, in particular colorectal cancer (CRC), as the risk of CRC among patients with CF is increased by 6-fold [5,6]. Moreover, there is an even stronger risk of CRC in patients with CF who have undergone a lung transplant [7]. Colonoscopy is considered to be the most effective method for the screening of CRC in patients with CF. Screening is started by the age of 40 for patients who have not undergone an organ transplantation; for transplanted patients the screening could start by the age of 35 or earlier [8]. This review is focused on the association of CF and CFTR with GI cancer. Furthermore, there is a discussion of the emerging therapeutic potential of modulator drugs in the prevention and treatment of CRC, an example of personalized cancer medicine.

## 2. Role of CFTR in Normal Physiological Conditions and Its Dysfunction in CF

The CFTR protein belongs to the ABC (ATP-binding cassette) class of transporters which are generally involved in transmembrane transport. The *CFTR* gene is transcribed into an mRNA transcript of 6128 nucleotides, which is then translated into a protein of 1480 amino acids. The CFTR protein is composed of two symmetrical halves with each half consisting of six membrane spanning domains joined by regulatory regions. The membrane spanning domains constitute an aqueous pore-like structure via which the Cl^−^ and HCO_3_^−^ ions are transported across the plasma membrane down their electrochemical gradient. Ionic specificity is determined by the amino acids which line the pore-like structure. Pore opening and closure is usually controlled by the binding of ATP to the nucleotide binding domains present in the regulatory region. The flow of ions through this channel results in the development of an osmotic pressure, which drives the movement of water in the same direction [9]. In the intestine, CFTR protein is expressed at the apical surface of epithelial cells throughout the entire length of the intestine. In the small intestine, the expression of CFTR gradually decreases from the duodenum to the ileum. In the colorectum, a high expression of CFTR is observed in the proximal colon and cecum. In both the small and large intestine, the highest expression of CFTR is observed in the crypts where the intestinal stem cells are present [10,11].

Biallelic inactivating mutations in the *CFTR* gene result in the development of CF. The most severe clinical manifestations associated with CF are pulmonary inflammation and obstruction, ultimately resulting in pulmonary failure. However, the dysfunction of CFTR in extra-pulmonary tissues, such as the pancreas and biliary ducts, accounts for CF-linked diabetes and liver diseases [12]. Loss of CFTR in the intestine results in obstruction in proximal colon and ileum in infants and distal intestine in older patients. Moreover, patients with CF are also prone to develop celiac disease. CFTR dysfunction in the GI tract results in low intestinal pH, thick mucus deposition, and an impaired innate immune response. These mechanisms generally drive the local inflammation of the GI tract that is proposed to increase the likelihood of an early onset of GI cancers. Furthermore, CF associated GI malfunction, along with antibiotic treatments, result in a significant alteration in microbial diversity of the gut, which is referred to as dysbiosis. A frequent lowering in the abundance of protective bacterial species such as *Acinetobacter Iwoffii* and *Lactobacillus* species with a corresponding increase in *Bacteriodes fragilis* and *mycobacterial* populations is observed in a CF gut. Alteration in microbial diversity enhances the pro-inflammatory effect associated with CF and dysbiosis and has been implicated in CRC development [9,11,13].

## 3. Involvement of CFTR in GI Cancers

### 3.1. Esophageal Cancer

Several studies have reported that CFTR dysfunction is associated with the development and progression of esophageal cancers, both esophageal squamous cell carcinoma (ESCC) and esophageal adenocarcinoma (EAC), and all studies support a tumor suppressing role for CFTR. Li et al. reported that CFTR protein expression was significantly lower in esophageal tumor tissues and esophageal cancer cell lines. They also reported that knockdown (KD) of CFTR in ESCC cells promoted the invasive behavior of these cells in vitro while also promoting ESCC tumor growth in vivo in xenograft mouse models. In contrast, CFTR overexpression in ESCC cells resulted in the suppression of cell proliferation, along with an increase in apoptosis. Silencing of CFTR was also found to promote the expression of NF-κB-p65 and NF-κB-p50 in ESCC cells. The effects of CFTR silencing in promoting cancer invasiveness was found to be reversed in the presence of NF-κB inhibitors, thereby highlighting the regulation of this pathway by CFTR [14]. The findings of Li et al. were supported by those of Matsumoto et al. who reported that CFTR overexpression in ESCC cells suppressed cell proliferation, migration and invasiveness while promoting apoptosis. Matsumoto et al. also carried out immunohistochemistry (IHC) analyses of the invasive front of primary tumor samples from ESCC patients and reported an inverse relationship between CFTR protein expression and post-operative survival. Finally, they reported the results of global gene expression microarray studies of KYSE 170 ESCC cells transfected with a CFTR-expressing plasmid showing that the p38 MAPK pathway played a crucial role in CFTR-mediated suppression of oncogenic phenotypes [15]. An additional confirming study was recently reported by Shi et al., who found that CFTR protein expression was downregulated as part of an autophagy-related gene (ARG) prognostic risk signature in ESCC patients [16]. For CF patients, studies have reported an increased risk of esophageal as well as gastroesophageal junction adenocarcinoma [5,6]. Adults with CF also have ~3-fold higher risk of developing Barrett’s esophagus and related neoplasms compared to individuals who did not have CF [17]. The findings of Knotts et al. were consistent with a prior genome-wide association study (GWAS) that reported that the CFTR locus was a risk locus for both Barrett’s esophagus and EAC [18]. Association of CFTR with esophageal cancer has been summarized in Table 1.

### 3.2. Pancreatic Cancer

Pancreatic insufficiency due to pancreatic damage, loss of acinar cells, fatty replacement and interstitial fibrosis is a common problem in CF [20]. It is well known that chronic inflammatory pancreatitis (CP) is linked to an increased risk for pancreatic cancer. While the incidence of CP in patients with CF is relatively low (<2%), there are several studies that have found an association between CFTR mutations and both CP and pancreatic cancer. Hamoir et al. reported that mutations in the *CFTR* gene were linked to genetically determined CP and pancreatic cancer. They also found that pancreatic cancer was diagnosed at an earlier age in subjects with CFTR mutations [21]. This finding was consistent with that of McWilliams et al., who also reported a modest enhanced risk of pancreatic cancer diagnosed at an earlier age in patients whose tumors carried CFTR mutations [22]. Maissoneueve et al. also reported a higher risk of early pancreatic cancer in patients with CF [5,6]. Several studies have also found that germline variants in the *CFTR* gene are linked to both pancreatitis and an increase in pancreatic cancer risk, including familial pancreatic cancer [23,24]. A separate study proposed that interactions between CFTR polymorphisms with environmental factors might be responsible for the development of pancreatic cancer [25]. Finally, CFTR has been reported to regulate the expression of MUC4, a glycoprotein implicated in tumor progression. Silencing of CFTR was found to enhance the expression of MUC4 in pancreatic cancer cells. Moreover, their expression profile demonstrated a negative correlation in these cells [26].

### 3.3. Hepatic Cancer

CF-related liver disease is a significant cause of death in patients with CF. Patients with CF also have a higher risk of hepatocellular carcinoma (HCC). In a case study conducted by Kelleher et al., an 18 year-old female with CF was diagnosed with advanced HCC, although she had no prior symptoms of chronic liver disease [27]. In another case study, a liver ultrasound performed on a 32-year-old female who was diagnosed with CF-associated liver disease reported lesions that were consistent with HCC. This was further confirmed by histological analysis [28]. In patients without CF, the downregulation of CFTR expression was reported in a study by Chen et al., who studied abnormally methylated differentially expressed genes in HCC, using gene expression profiles available from the Gene Expression Omnibus (GEO) that identified CFTR as a hypermethylated, low expressing hub gene in HCC [3]. Similar downregulation of CFTR by promoter hypermethylation was reported by Moribe et al. in a study of 25 HCC samples compared with normal control tissues [29]. CFTR also plays an important role in the biliary tract, specifically in biliary epithelial cells (cholangiocytes), where it is expressed on the apical membrane. Maisonneuve et al. reported a higher risk of biliary tract cancers in patients with CF [5,6]. There have been at least two studies indicating that CFTR acts to prevent proinflammatory pathway activation that is associated with susceptibility to cancers in the biliary tract, such as cholangiocarcinomas. Fiorotto et al. reported that CFTR controlled biliary epithelial inflammation by regulating Src tyrosine kinase activity, and further prevented phosphorylation of TLR4 and NF-κB activation [30]. Hu et al. identified a CFTR-β-catenin-NF-κB interactome, whereby loss of CFTR in cholangiocytes resulted in NF-κB activation and inflammation [31].

### 3.4. Gastric Cancer

To date there has not been a strong association between CFTR dysfunction and a risk for gastric cancer, either in patients with CF or in patients without CF. There is a single report that studied 1468 individuals who were heterozygous germline carriers of F508del mutations that found an increased risk of gastric cancer in these individuals (O.R. 2.5 [CI 1.6–3.4]) [32]. There is also one report that serum CFTR levels correlated with the serum expression of the tumor biomarker CA199 in gastric cancer [33]. See Table 2 for a summary of the association of CFTR with Pancreatic, Hepatic and Gastric cancers.

### 3.5. Intestinal Cancer

CFTR mutations or the downregulation of CFTR expression has been strongly implicated in the development and progression of intestinal cancers of both the small and large bowel, particularly colorectal cancer, in patients with CF and patients without CF and in animal models of CFTR-deficiency. Indeed, CFTR’s action as a tumor suppressor in the GI tract is considered strongest in the intestine. Maissoneuve et al. reported a 6-fold increased risk of CRC in CF patients based on a 20-year epidemiological study [5,6]. Since then, follow up studies have further supported a strong role for CFTR in prevention of CRC. Yamada et al. carried out a meta-analysis of 6 population-based studies involving more than 99,000 patients and confirmed a high risk of GI cancers in patients with CF [7]. Endoscopic screening studies of adult patients with CF showed a high incidence of early aggressive tumors beginning in their 30s, with 50% of CF patients having developed colorectal tumors by age 40, with some having advanced to adenocarcinomas [34,35]. CF has now been identified as a hereditary colon cancer syndrome by the Cystic Fibrosis Foundation with the recommendation that CRC endoscopic screening begin in patients with CF by age 40, and in immunocompromised lung transplant patients with CF, at particular risk for cancer, by age 30 [36]. Finally, CFTR’s role as a tumor suppressor in intestinal cancer was confirmed in an intestinal specific CFTR knockout mouse model. Mice carrying a floxed allele of CFTR exon 10 were crossed to mice expressing the intestinal-specific villin-Cre transgene. These CFTR KO mice were then introgressed into the *Apc^Min^* mouse model of intestinal cancer, resulting in the significant enhancement of tumorigenesis throughout the intestinal tract compared with *Apc^Min^* CFTR wildtype littermate control mice. Of further interest, >60% of mice that were mutant for CFTR alone (*Apc* wildtype) developed intestinal tumors when aged to one year, a phenotype not observed in CFTR wildtype mice [37].

CFTR germline carriers, of which there are ~10 million individuals in the US alone, are also at risk for CRC. A population-based study of more than 19,000 heterozygous CFTR mutant carriers and 99,000 healthy controls found that CF carriers were at an increased risk for 59 CF-related diagnostic conditions, including a 44% increased risk of GI cancers (colorectum, stomach and other GI organs) [38,39]. In a separate study of over 79,000 cancer patients in the UK, using data from the UK Biobank, Shi et al. found that heterozygous carriers of the F508del mutation had an overall higher risk of all cancers, and were specifically at a higher risk of CRC and gall bladder and biliary cancers. For patients with pancreatic cancer, there was an enhanced risk that did not reach significance [40]. In addition to these findings in heterozygous carriers of known pathogenic CFTR mutations, there have also been reports of heterozygous CFTR polymorphic variants linked to CRC risk. Notably, there has been a marked increase in the incidence of CRC in young adults throughout the western world and a study of 133 young adults with CRC identified 25 associated polymorphic variants. Almost all of these 25 variants were in known DNA repair genes such as MLH1, MSH2, MSH6, MUTYH, ATM and BRCA2. However, 2 of the 25 variants were found in CFTR [41]. Another recent study identified 14 polymorphic variants involved in familial predisposition to serrated polyposis syndrome, a condition in which colorectal polyps are highly susceptible to progression to adenocarcinomas. Of the 14 polymorphic variants identified, 2 of the 14 were found in CFTR [42].

There is also strong evidence that CFTR acts as a tumor suppressor in sporadic, non-CF CRC, lacking germline CFTR mutations. Than et al. reported that stage II CRC patients whose cancers maintained high CFTR expression had a significantly better prognosis and disease free survival (DFS) than patients whose cancers demonstrated low CFTR expression [37]. Sun et al. reported that CFTR mRNA and protein expression was lower in CRC tumors vs. normal tissues and CFTR mRNA expression was lower in metastatic CRC vs. non-metastatic CRC. Furthermore, Sun et al. found that CFTR-depleted CRC cell lines had a more oncogenic phenotype, including increased invasion, migration and colony formation [43]. Moreover, CFTR has also been found to influence the proliferation of intestinal cells. Increased proliferation was observed in a CF KO mouse intestine and CFTR silenced CaCO_2_ cells. CFTR-mediated regulation of cell proliferation was associated with modulation of hedgehog signaling [44]. Liu et al. reported similar results of the downregulation of CFTR expression mRNA and protein in a study of 70 human CRC samples, as measured by qRT-PCR and IHC [45]. CFTR deficiency has also been implicated in cancers of the small intestine [9], including in patients with CF [5,6], a finding that is consistent with CFTR’s key role as an anion transporter in regulating water, fluid and bicarbonate transport in the small intestine, such as in the Brunner’s glands. See Table 3 highlighting the involvement of CFTR in Intestinal cancer.

## 4. Potential Molecular Mechanisms Involved in the Regulation of CFTR in Cancer

CFTR has been found to act as a tumor suppressor in various cancer types and its expression is generally downregulated in tumors. There are different mechanisms which have been implicated in the regulation of CFTR expression. Much of the evidence for these mechanisms has been generated in studies in the intestinal tract, so it is very possible that novel mechanisms of CFTR regulation may yet be discovered in non-intestinal tract GI cancers.

### 4.1. Epigenetic Regulation

#### 4.1.1. Promoter Hypermethylation

The expression levels of CFTR mRNA and protein were found to be silenced by epigenetic mechanisms, mainly hypermethylation of the CFTR promoter, in several of the major cancers where CFTR has been shown to act as a tumor suppressor. Liu et al. reported that CFTR expression was silenced in CRC. Methylation specific PCR (MSP) was performed to determine the promoter methylation status of CFTR in CRC tissues. Results of the MSP analysis found that ~62% of CRC patient tissue samples demonstrated hypermethylation of the CFTR promoter. Furthermore, CFTR promoter methylation status was found to be significantly correlated with age and lymph node metastasis [45]. Elsewhere in GI tract cancers, similar epigenetic modifications were found to modulate CFTR expression in HCC [29]. CFTR promoter hypermethylation and gene silencing were also found in cancers outside the GI tract. Using MSP, Shin et al. found that the CFTR promoter was hypermethylated and CFTR expression was downregulated in head and neck cancer (HNC) compared with normal tissues. This was further supported by the results of bisulfite sequencing, which clearly indicated that CFTR CpG islands were more methylated in the HNC tissues compared to normal controls [47]. CFTR levels were also found to be silenced by promoter hypermethylation in lung adenocarcinoma (LUAD) [48,49], lung squamous cell carcinoma [50], breast cancer [51] and prostate cancer [52].

#### 4.1.2. Activity Involving CIS-Regulatory Elements (CREs)

Three-dimensional chromatin structure analysis revealed another epigenetic mechanism, *cis* regulatory elements (CREs) encoding for enhancers at the CFTR locus, that have been reported to regulate *CFTR* gene expression in intestinal and airway epithelial cells. CFTR lies within a topologically associated domain (TAD) flanked by CTCF and cohesion occupancy [53]. Within this TAD, specific CREs play a key role in the recruitment of activating factors to the CFTR promoter. In work carried out by the group of Ann Harris in CaCO^−2^ CRC cells, CRISPR-Cas9 was used to remove CREs, individually or in tandem. Subsequent assays for monitoring gene expression and an analysis of higher order chromatin structure (4C-seq) highlighted the interaction between two cell type specific intronic enhancers, with loss of these enhancers resulting in an ablation of CFTR expression. Moreover, this mechanism, which is activated when the CREs are lost, involves the loss of CFTR activating transcription factors, including FOXA2 [54], and the loss of higher order chromatin structure. Furthermore, studies by the group of Stephanie Moisin identified four intestinal-specific CREs that are critical for regulation of CFTR expression in intestinal cells [55].

#### 4.1.3. Silencing by MiRs

Another epigenetic mechanism that has been shown to modulate CFTR expression are miRNAs (miRs). An example is miR-125, which has been reported to be upregulated in primary CRC tumors in addition to metastases. In vitro, the overexpression of miR-125 in CRC cells enhanced invasion and migration. MiR-125 was shown to target CFTR, as supported by the results of a dual-luciferase assay. The targeting of CFTR by miR-125 enhanced the expression and the secretion of the urokinase plasminogen activator (uPA), along with the promotion of epithelial to mesenchymal transition (EMT) [56]. Another miR, hsa-miR-1246, showed involvement in the regulation of CFTR in CRC cells. Inhibition of this miRNA was found to elevate the protein levels of CFTR in both CaCO^−2^ and SW620 cells. To elucidate the mechanism of regulation, the 3′ UTR of CFTR was cloned into a luciferase vector. The reduction in luciferase reporter activity upon the transfection of hsa-miR-1246 mimics indicated that hsa-miR-1246 represses the expression of CFTR by binding to the 3′UTR binding site (position 1537–1544 of 3′UTR) [57].

#### 4.1.4. Action of Transcription Factors

Krüppel-like factors (KLFs) are a family of transcription factors (TFs) involved in development, proliferation and stem cell differentiation. Among them, KLF4 was found to be upregulated in CF respiratory epithelial cells compared to non-CF cells. Moreover, KLF4 overexpression negatively regulated the expression of CFTR in cells expressing wildtype CFTR. However, in the case of cells expressing F508del mutants, KLF4 had no impact on CFTR levels [58]. Another member of this TF family, KLF5, repressed the expression of CFTR in human primary airway epithelial cells and cell lines [59]. The depletion of KLF5 altered the higher order chromatin organization of the CFTR locus, thereby affecting its expression. Critical looping interactions, which are required for normal CFTR expression, were altered along with the redistribution of the H3K27ac active chromatin mark [60]. The KLF family of transcription factors, particularly KLF4 and KLF5, have been reported to promote GI cancer progression [61]. Therefore, KLF4/KLF5 may also be involved in the downregulation of CFTR expression in GI cancers.

As discussed above, CFTR-specific CREs at the CFTR locus play critical roles in the regulation of CFTR expression. Specific TFs that bind CFTR CREs can either promote or repress CFTR expression. NandyMazumdar et al. reported that BACH1, a master regulator TF of oxidative stress, can bind to CFTR CREs and either repress or activate CFTR, depending on whether a cell is under normoxic conditions or under hypoxia and/or oxidative stress. Another differential context was whether the cells were from airway respiratory epithelial cells or intestinal epithelial cells. In airway cells under normoxic conditions, BACH1 occupies a distal -44kb antioxidant response element (ARE) located in a CRE and CFTR expression is repressed, while under hypoxic conditions, NRF2 displaces BACH1 from the ARE, permitting the activation of CFTR. Of added interest, BACH1 has shown that it can independently and indirectly activate CFTR by a mechanism that is not clearly understood [62].

### 4.2. Altered Signaling Pathways

CFTR expression and functioning has been shown to be regulated upstream by various signaling mechanisms, including the AKT and GSK3β pathways. These two pathways act in an opposing manner to regulate CFTR expression. AKT acts as a positive regulator while GSK3β negatively regulates CFTR expression [58]. CFTR has also been shown to be involved in the regulation of various downstream signaling pathways, including NF-κB and ERK in tumor cells [14,63].

## 5. Potential Mechanisms by Which CFTR Deficiency Promotes Carcinogenesis in the GI Tract

Given that much of the mechanistic research into CFTR deficiency in cancer has focused on the intestinal tract, evidence for potential causative downstream mechanisms largely still comes from that tissue, with potential applicability to other GI tract cancers and/or the discovery of novel mechanisms in non-intestinal GI tract cancers.

### 5.1. Influence on the Stem Cell Compartment

CFTR is generally expressed in the mucosal epithelia of the esophagus, stomach and both the small and large intestine. In liver, CFTR is primarily expressed in the intrahepatic biliary epithelium, and in the pancreas, CFTR expression is strongest in the small, intercalated ducts. Here, it is important to acknowledge that while CFTR expression has traditionally been described in the epithelia of tissues throughout the body, recent reports have demonstrated a much broader expression pattern for CFTR, for example, in the brain. Additionally, in the GI tract, CFTR has also been found to be expressed by enteric ganglia and in a variety of non-epithelial cells, such as fibroblasts, endothelial cells, neutrophils, lymphocytes, macrophages and mast cells, where its functions in these various cell types are yet to be characterized. Nonetheless, overall, in the GI tract, CFTR expression is strongest in the mucosal epithelia of the small and large intestine, principally in the crypts and localized to the base of the crypts that contain the intestinal stem cell compartment. Outside of the intestinal tract, much less is known about the identity and function of resident tissue stem cells. In the intestine, CFTR expression in the crypt has been reported in the stem cell compartment, or immediately adjacent to it [46]. Thus, CFTR is well placed to influence intestinal crypt renewal and the activity of putative cancer progenitor cells. For example, CFTR has been reported to influence intestinal stem cell lineage differentiation [64]. Intestinal stem cell capacity can be modeled using 3D organoids that retain normal crypt structure, polarity and physiology [65]. CFTR function has been tested in colon organoids created from both humans, including patients with CF, and in mouse models, and CFTR has been shown to retain its normal ion (Cl^−^, HCO_3_^−^) activity in the organoids [66,67]. Notably, colon organoids from CF patients have been used as valuable surrogates for the testing of therapeutic modulator drugs (as discussed below) [68,69,70]. Organoids can also be used to measure the oncogenicity and response to therapy of patient-derived cancerous tissues [71,72]. Organoids created from CFTR KO mice demonstrated increased clonogenicity [37], along with localization of CFTR to the leucine rich repeat containing G protein-coupled receptor 5-posiitive (LGR5+) intestinal stem cells in the small intestine [46].

### 5.2. Regulation of Wnt/β-Catenin Signaling

Wnt/β-catenin signaling is an essential mediator of intestinal tissue homeostasis, including stem cell survival, proliferation and differentiation, functions that are critical for normal intestinal cell renewal. Dysregulation of the Wnt/β-catenin signaling pathway is involved in ~90% of human CRC, both in early tumor initiation and in progression to invasive cancer [73]. CFTR deficiency in mouse small intestine was found to be linked to increased Wnt/β-catenin signaling [37] and tumors isolated from the small intestine of one year-old conditional intestinal-specific CFTR KO mice (*Apc* wildtype) had enhanced nuclear localization of β-catenin, as determined by IHC, along with elevated expression of Wnt/β-catenin target genes, such as *Cyclin D1* (*Ccnd1*), *Lgr5* and *cluster of differentiation 44* (*CD44*), as determined by RNA-Seq and q-RT-PCR [37]. This finding was confirmed in a separate study using CFTR constitutive KO mice, where KO mice showed enhanced intestinal stem cell proliferation and a significant increase in Wnt/β-catenin signaling [46]. Outside of the intestinal tract, Wnt/β-catenin signaling is also dysregulated in all of the other major GI tract cancers, including esophageal cancer [74], gastric cancer [75], liver cancer [76], and pancreatic cancer [77]. However, to date there have been no reports yet linking CFTR dysregulation to aberrant Wnt/β-catenin signaling in these cancers. However, given the evidence linking CFTR dysregulation and Wnt/β-catenin signaling in the intestine, including in the stem cell compartment, it will not be surprising if these connections between CFTR and Wnt/β-catenin signaling in non-intestinal GI tract cancers emerge from ongoing studies.

### 5.3. Disruption of Physical Barriers and Microbial Dysbiosis

CFTR is involved in multiple physical processes in the intestine that are critical for the maintenance of tissue homeostasis, including physical barriers to protect the single cell epithelial layer, the gut microflora and management of the innate and adaptive immune responses. Disruption of these processes is associated with tissue damage, inflammation and the creation of a favorable landscape for cancer development [78]. Importantly, there is evidence of gut inflammation in both patients with CF and in CF mouse models [79,80]. Further consistent with a role for CFTR in the maintenance of intestinal homeostasis is the report of its downregulation in ulcerative colitis [81].

Two major physical barriers protect the colonic epithelium from contact with the microflora. First, the apical surface of the epithelium is covered by a dense inner and looser outer mucus layer. The loose outer layer contains abundant commensal bacteria, along with the potential presence of pathogenic bacteria [82]. The largely sterile inner layer is dependent on mucin2 (MUC2) proteins that are secreted by goblet cells and that, in turn, are dependent on bicarbonate ions (HCO_3_^−^) and water [83]. CFTR is directly involved in HCO_3_^−^ efflux into the lumen [84] and also indirectly involved in H_2_O efflux [85,86]. CFTR deficiency causes the mucus layer to become dehydrated and dysfunctional. In the distal small intestine of children with CF, this causes an obstructive disorder called meconium ileus [87,88]. Obstruction in the proximal colon in both humans and mouse models causes illicit bacterial contact with the epithelium and consequent inflammation that can predispose to cancer. CFTR has also been reported to be involved in MUC2 goblet cell exocytosis dysfunction [89]. Than et al. employed GSEA to compare gene expression profiles between the colons of CFTR KO and *Muc2* KO mice and reported an enrichment in inflammatory gene expression [37], supporting that CFTR deficiency, similar to *Muc2* deficiency, allows dysregulated bacterial contact with the epithelial layer. A second protective barrier in the epithelial layer consists of the tight junctions between cells that are in constant breakage and reestablishment as cells migrate up and out of the intestinal crypt. It was shown that CFTR deficiency in the small intestine of CFTR KO mice resulted in increased epithelial permeability and disruption of tight junctions, with evidence that CFTR may mediate tight junction integrity via its C-terminal PDZ-binding domain [90]. Other reports indicate that CFTR deficiency could disrupt tight junctions by preventing its protein–protein interactions with TJP1 (tight junction protein-1)/ZO-1 (zona occludins 1) [91].

Bacterial dysbiosis and associated inflammation is commonly found in the gut of both human patients with CF and in CFTR KO mice [13,87,92,93,94,95,96,97]. The link between CFTR deficiency and microbial dysbiosis is consistent with the key role that intestinal ion transport has on the composition of gut microbiota [98]. Indeed, low grade inflammation and changes in the gut microbiome are now considered hallmarks of a CF intestine [78]. The severity of microbial dysbiosis has also been linked to the severity of CFTR mutations with the most severe dysbiosis observed in patients with CF-harboring mutations such as F508del [99]. Microbial dysbiosis is also a common feature in CRC [100], thus CF-related dysbiosis may play an important role in susceptibility to CRC [101]. Large scale sequencing of gut microflora in human patients with CF and in CF mouse models have revealed significant changes in bacterial diversity, richness and the presence and absence of protective vs. harmful bacterial species, with potentially oncogenic consequences [97,102,103]. While much of the damage that occurs as a result of microbial dysbiosis involves changes in the relative composition of resident commensal bacteria, these changes can also create niches for the introduction and spread of pathogenic species. The presence of pathogens has also been reported to negatively affect CFTR functions such as barrier protection, as shown in an in vitro study using human organoid-derived intestinal cultures infected with *Giardia duodenalis* that caused a reduction in CFTR-dependent chloride secretion and a breakdown in tight junction structure [104]. Changes in gut flora can also lead to changes in host gene regulation. Dayama et al. compared colonic mucosal samples between patients with CF and healthy controls, analyzed by RNA-seq and 16S rRNA sequencing, and described interactions between changes in the gut microbiome and host gene expression, identifying more than 1500 host genes differentially expressed in the colon of patients with CF [105].

### 5.4. Proinflammatory Immune Cell Infiltration and Proinflammatory Signaling

Disruption of physical barriers not only permits access of bacteria to the epithelial layer of cells but also leads to infiltration of immune cells of the lamina propria into the epithelial layer, resulting in tissue damage and inflammatory signaling, mediated by the release of inflammatory cytokines by innate immune cells and the recruitment of proinflammatory cells of the adaptive immune response. Immune cell infiltration has been observed in patients with CF and in CF KO mouse models but clear histological evidence of tissue damage has not been observed [106]. Recent work has also highlighted the role of CFTR in various cells of both the innate and adaptive immune response, such as lymphocytes and NK cells [107,108]; thus, CFTR deficiency in immune cell populations may cause aberrant immune cell activity, as has been reported for several cell types of the innate immune response [109]. For example, neutrophils in patients with CF have been shown to have intrinsic impairment linked to degranulation [110]. Another example was the rescue of monocyte recruitment and improvement in overall health in constitutive CF KO mice following bone marrow transplantation from CFTR wildtype donor mice [111].

CFTR deficiency is associated with the activation of proinflammatory signaling pathways that can promote cancer development. Activation of NF-κB signaling pathways has been observed downstream of CFTR deficiency in several tissues and cancers including in esophageal and intestinal cancers [45,112,113,114,115,116]. The expression of proinflammatory cytokines has been reported in several CFTR-deficient human CRC cell lines, including CaCO^−2^ and HT-29 cells [114,117,118,119], and outside the GI tract CFTR has been shown to regulate the proliferation, migration and invasion of cervical cancer cells via inhibition of NF-κB signaling [120]. These numerous studies reported the upregulation of many notable NF-κB pathway members, including the upregulation of tumor necrosis factor alpha (TNF-α), interleukin-6 (IL-6), and interleukin-1-β (IL-1β)-induced secretion of interleukin-8 (IL-8), cyclooxygenase-2 (COX-2) and prostaglandin E2 (PGE_2_), along with an enhancement in the activity of extracellular signal-related kinases 1/2 (ERK1/2), mitogen-activated protein kinase (MAPK), NF-kappa-B inhibitor alpha (IκBα), and NF-κB. Massip-Cortez et al. also reported that CFTR deficiency caused activation of NF-κB following autocrine signaling by IL-1β [121].

### 5.5. Altered Stress Responses

Due to high rates of metabolism in cancer cells, including CRC, oxidative stress and the production of reactive oxygen species (ROS) is increased. ROS can promote cancer development via damaging DNA, causing mutations, but ROS is also stressful for both normal and cancer cells, which can promote apoptosis. Several studies have reported that CFTR deficiency in normal cells is linked with an increase in cellular oxidative stress associated with mitochondrial dysfunction and an increase in ROS [122], and, conversely, a reduction in cellular oxidative stress by cellular retention of the antioxidant glutathione (GSH) [123]. KD of CFTR in human CaCO^−2^/15 CRC cells caused an increase in lipid peroxidation and catalase [117]. CFTR-deficiency has also been associated with the downregulation of cellular autophagy caused by transglutaminase-2 (TG2) [124].

A pathway of particular interest in the regulation of CFTR in oxidative stress is the HIF-1/NRF2 pathway. In unstressed cells NF-E2-related factor 2 (NRF2) is sequestered in the cytoplasm via binding to Kelch-like ECH-associated protein 1 (KEAP-1), leading to its ubiquitination and proteosomal degradation. In response to cellular stress, such as under hypoxia and increased levels of ROS, NRF2 is translocated to the nucleus where it binds to AREs and activates target genes. However, importantly, this response appears to be tissue specific. There are several reports that NRF2 can either positively or negatively regulate CFTR expression, depending on whether the cells are under normoxic or hypoxic conditions, whether the cells studied are airway epithelial cells vs. intestinal epithelial cells (or renal cells), and whether the cells are normal vs. malignant, and possibly whether the malignant cells are early stage vs. late-stage cancer [125]. Thus, the actions of NRF2 on CFTR expression appear to be context dependent. Furthermore, NRF2 expression has been reported to both promote and prevent cancer development, although there is more of a consensus, based on studies of multiple human cancers, that NRF2 expression is a poor prognostic factor [126].

Several groups have reported that NRF2 is downregulated in patients with CF and CF KO mouse models [127], a phenotype associated with an increase in ROS and an increase in inflammatory pathways such as NF-κB. As discussed above, the CFTR locus lies within a TAD that contains CREs, including a -44kb distal CRE that harbors an ARE. This -44kb ARE is active in airway epithelial cells but not in intestinal epithelial cells [125]. Under normoxic conditions the -44kb ARE is bound by repressors, including BACH1 (and other repressors). Following oxidative stress such as treatment of cells with sulforaphane (SFN), a naturally occurring chemopreventative and anticancer agent [126] NRF2 has been shown to displace the repressor proteins at the -44kb ARE, leading to CFTR activation in airway epithelial cells [126], an activation that is accompanied by changes in chromatin architecture within the TAD. Interestingly, F508del corrector drugs have now been shown to rescue NRF2 dysfunction [128]. However, it should be noted that there are also contradictory reports that indicate that ROS activation of NRF2 and binding to AREs can also downregulate CFTR expression in airway epithelial cells. All of these studies have been carried out in various bronchial epithelial cell lines; thus, the choice of a particular airway epithelial cell line may be relevant, another possible example of context dependency for the effect of NRF2 on CFTR. Given that much of what is known about the regulation of CFTR expression under conditions of oxidative stress and increases in ROS is based on studies in airway epithelial cells, there remains a significant gap in understanding what may be happening in intestinal epithelial cells, especially CRC cells. One possibility is that additional stress and NRF2 responsive CREs (in addition to the -35kb and -44kb CREs) may mediate effects in the intestine, such as the four novel CREs recently described by Collobert et al., that lie within introns of CFTR [55].

There are links between gut ischemia, hypoxia, dysbiosis [129] and inflammation. For example, severe burns can cause a breakdown of intestinal epithelial barriers, inflammation, bacterial translocation and dysbiosis. A study in CaCO^−2^ CRC cells under hypoxic conditions found a significant downregulation of CFTR expression and an increase in levels of ERK1/2 and NF-κB signaling and related inflammatory factors such as TNFα, IL-1β, and IL-8. KD of CFTR in CaCO^−2^ cells produced a similar phenotype [130]. This is consistent with reports that CFTR mRNA and protein expression and function has been reported to be repressed by HIF-1 in hypoxic epithelium, including T84 and CaCO^−2^ CRC cells, which is common in most epithelial cancers, including CRC [130,131].

How these sometimes-contradictory stress responses and their connections to CFTR influence carcinogenesis remains unclear, and they may well be tissue and cancer context-dependent, and therefore the role of CFTR in these tissues and cancers may also be context-dependent. One possibility is that loss of CFTR protects CRC cells from ROS-induced apoptosis via retention of antioxidants such as GSH. Preliminary work by our group indicates that loss of CFTR in CaCO^−2^ CRC cells promotes their survival following treatment with oxidative-stress-inducing agents such as menadione [132]. Similar results were observed in CFTR KO mouse colon organoids treated with menadione [133].

**Figure 1:**
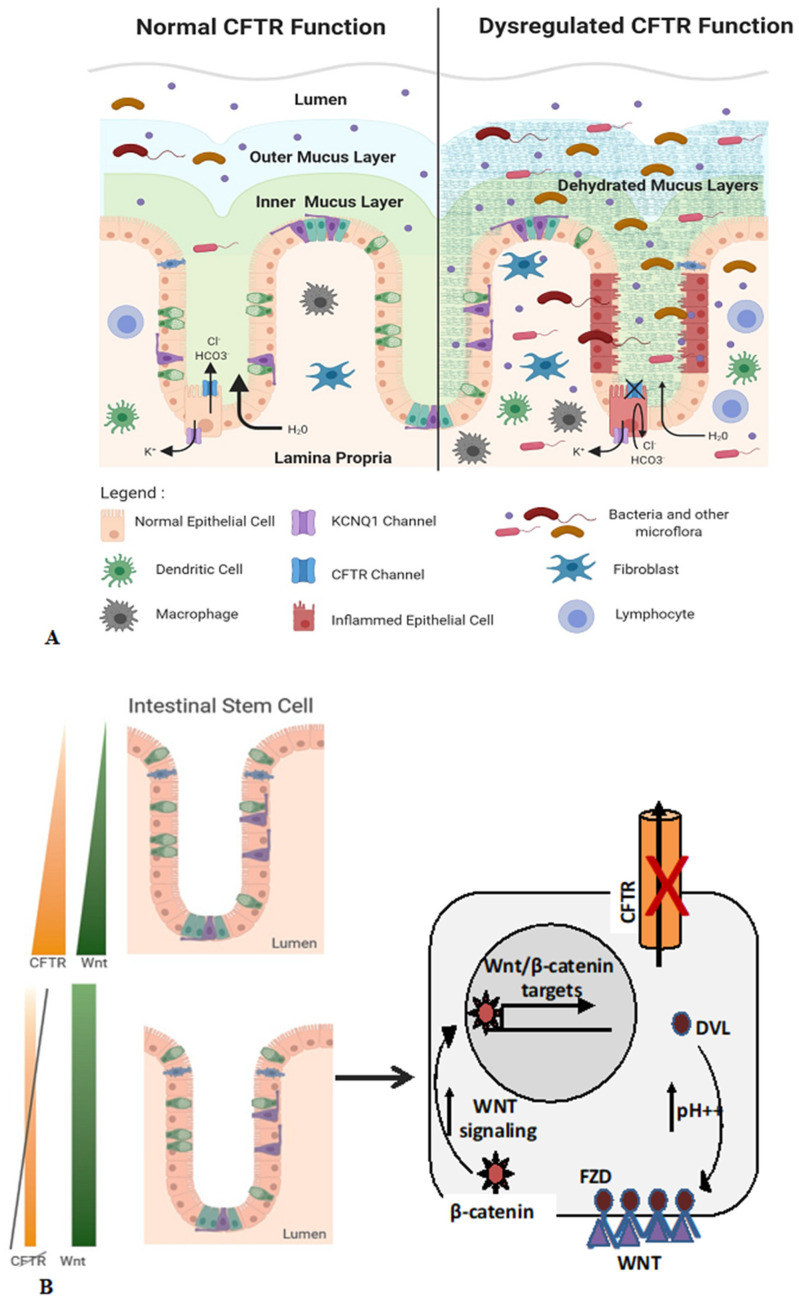

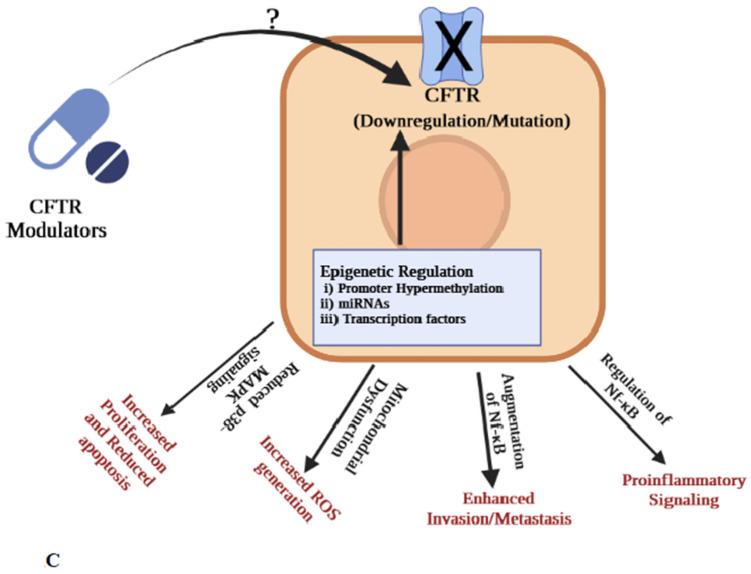
CFTR dysfunction in the GI tract and its association with carcinogenesis. (**A**) CFTR deficiency disrupts protective physical barriers resulting in microbial dysbiosis, inflammation as well as immune cell infiltration. Deficiency of CFTR results in the failure of intestinal chloride as well as bicarbonate ion transport and the accompanying efflux of water molecules. Loss of CFTR accounts for the dehydration of the mucus layer, making it permissive for bacterial infiltration and also results in intestinal obstruction. The disruption of epithelial barrier causes infiltration of pathogenic and commensal bacteria, inflammation, infiltration of immune cells and epithelial tissue damage. These alterations result in the generation of a favorable niche which promotes the initiation and progression of GI cancers [11]. (**B**) CFTR downregulation enhances Wnt/β-catenin signaling. Expression levels of CFTR is highest in the intestinal crypts, in particular at the base of the crypt in the stem cell compartment, with expression levels lower in the intestinal lumen. Wnt expression levels follow a similar gradient under normal conditions. Loss of CFTR activity in the crypts increases intracellular pH (pHi) and results in stabilization of the plasma membrane association of Disheveled (Dvl). This ultimately enhances Wnt/β-catenin signaling via the nuclear translocation of cytoplasmic β-catenin. Nuclear β-catenin promotes the expression of genes involved in survival as well as proliferation of intestinal stem cells, which increases the risk of intestinal tumor development. (**C**) CFTR dysfunction promotes carcinogenesis. Loss of CFTR function in a cell can occur due to genetic mutations or different epigenetic mechanisms including promoter hypermethylation, activity of certain transcription factors, changes in chromatin architecture as well as miRNAs, resulting in the downregulation of CFTR expression. Downregulation of CFTR results in increased cellular proliferation along with decreased apoptosis, increased invasive behavior, mitochondrial dysfunction resulting in ROS generation and increased proinflammatory signaling. These properties are mediated by specific signaling pathways, including the p-38 MAPK and NF-κB pathways. These alterations in cellular behavior promote cancer progression. As CFTR modulator drugs have shown efficacy in CF patients, they may also show promising results when used in cancer cells where the function of CFTR is lost. This figure was created using BioRender software.

See Figure 1 for a summary of the various potential anti-cancer mechanisms of CFTR.

## 6. Targeting Specific CFTR Mutations with Modulator Drugs, Implication for GI Tract Pathologies, including Cancer

CFTR modulator drugs function by either restoring or elevating the expression, stability and function of CFTR protein. These drugs now constitute an effective therapy for most patients with CF [134]. Based on their mode of action, the modulators have been divided into five main categories: potentiators, correctors, stabilizers, read-through agents, and amplifiers. R334W, R347P, and G551D are among the most common mutations that result in impaired CFTR channel gating or conductance. Potentiators are compounds that increase CFTR channel open probability, thereby enhancing anion conductance. Ivacaftor is a potentiator which was found to enhance CFTR conductance in cell lines with G551D mutations [135]. In 2012, both the US Federal Drug Agency (FDA) and European Medicines Agency (EMA) approved the use of ivacaftor (Kalydeco^®^, Vertex Pharmaceuticals) for the treatment of patients with CF (aged ≥6 years) who carry at least one G551D mutation [2]. The most common CFTR mutation (F508del) results in improper CFTR protein folding and trafficking. Correctors are a class of modulator compounds which promote proper folding, processing as well as trafficking of the CFTR protein. Lumacaftor (VX-809; Vertex Pharmaceuticals) is a first-generation corrector which has shown promising results in the rescue of CFTR protein folding and function in both primary bronchial epithelial cells and F508del-expressing cell lines. Both FDA and EMA (in 2015) approved the co-treatment of a lumacaftor/ivacaftor combination (Orkambi^®^, Vertex Pharmaceuticals) for patients homozygous for the F508del mutation and ≥12 years [136]. Another compound, tezacaftor, is a second-generation corrector which was developed based on lumacaftor structure. However, tezacaftor has demonstrated better pharmacokinetic properties along with lesser side effects [137]. Another class of modulators called stabilizers enhance the stability of CFTR protein, anchoring them to the plasma membrane and preventing its lysosomal degradation. Cavosonstat (N91115; Nivalis) was the first stabilizer tested in clinical trials. Apart from these mutations, a number of CFTR mutations account for the introduction of a premature termination codon (PTC) in the mRNA, thereby abrogating protein synthesis or leading to the generation of a truncated non-functional protein. Moreover, these mRNAs are also subjected to NMD (nonsense mediated decay), thereby reducing expression. G542X and W1282X are the most common PTC mutations that are found in patients with CF. A particular class of modulators known as the read-through agents cause ribosomal over-reading of a PTC, accounting for the incorporation of a foreign amino acid in place of the mutation, thereby allowing translation to continue until the end of the mRNA transcript [138]. Read-through effects were first observed in the aminoglycoside antibiotics, such as geneticin and gentamicin. A read-through agent, ELX-02, which is an aminoglycoside analog, has been tested in several volunteers in phase I clinical trials, where it was found to be well tolerated [139]. Moreover, the compound Ataluren was identified by HTS, and it restored the expression and function of CFTR in transgenic mice that expressed human G542X. However, this compound did not elicit any significant therapeutic effect in phase III clinical trials [2]. Mutations such as A455E reduce CFTR protein synthesis and maturation. Amplifiers are a class of modulators that elevate CFTR mRNA levels and, subsequently, protein synthesis. PTI-428 (nesolicaftor; Proteostasis Therapeutics) is the first-in-class amplifier that was investigated in clinical trials. This compound enhanced the expression of immature CFTR protein carrying different mutations [140].

The most common CFTR mutation is F508del, which is found in ~70% of patients with CF. For patients who are homozygous for this mutation, the combination of a single corrector (tezacaftor or lumacaftor) with a potentiator (ivacaftor) has been shown to improve clinical outcomes substantially. However, patients with CF who are heterozygous and have one F508del allele along with another mutation that accounts for non-responsiveness to these dual modulator combinations are referred to as “minimum function” mutations. For these patients, treatment with the next-generation CFTR corrector, elexacaftor, used in combination with tezacaftor and ivacaftor, known as Trikafta^®^ (VertexGPS), has been found to show promising results in improving CFTR function and clinical outcome [141].

Importantly, CFTR protein function modulators have also shown promise in the treatment of CF-associated GI pathologies [142,143,144,145]. A key reagent for the preclinical testing of these drugs has been the development of patient-derived organoids from the colorectum [67,69]. The CFTR potentiator ivacaftor was found to modulate intestinal pH as well as the microbial population. Treatment of this compound resulted in the alteration of the gut microbiome and also reduced inflammation in patients with one copy of a CFTR gating mutation [146]. This effect on improving fecal microbiota via ivacaftor treatment was especially effective in patients with CF with pancreatic insufficiency [147]. Furthermore, ivacaftor based therapy was found to reduce the frequency and the recurrence rate of pancreatitis in CF patients [148]. Owing to the fact that many patients with CF are at a high risk of developing GI cancers, especially CRC, these modulators might prove to be beneficial for the treatment of those cancer patients with specific CFTR mutations. This modulator-based treatment regimen might prove to be a more specific and precise mode of cancer therapy. CFTR modulator drugs may also act as chemopreventative agents. With the growing numbers of patients with CF receiving one or more modulator drugs, primarily to treat lung disease, it will be interesting to see if the frequency of various early onset GI cancers is reduced, especially for the incidence and aggressiveness of colon tumors.

Apart from these modulator compounds, other molecules have also been found to be effective in regulating the expression as well as the functioning of mutant CFTRs. The RNA-binding protein (RBP), splicing factor proline/glutamine-rich (SFPQ) is generally associated with the regulation of gene expression and intracellular trafficking. SFPQ was found to elevate the expression along with the function of CFTR F508del mutants by modulating different signaling pathways in lung epithelial cells [149]. Another potential molecule-based therapy for CF is thymosin α1 (Tα1), a naturally occurring polypeptide with an excellent safety profile that has been shown to rectify multiple CF tissue defects in CF KO mice and tissues from patients with F508del mutations [150]. Finally, CFTR also appeared to be a molecular target for the anthraquinone compounds obtained from laxative herbal plants. These compounds acted as CFTR potentiators, which enhanced chloride as well as fluid secretion from the colon [151]. It should be noted that the potential effect of CFTR modulators in suppressing tumor aggressiveness is still a hypothesis and, thus, the outcome of these modulator drug treatments in cancer patients needs to be monitored for the development of future treatment regimens based on them.

## 7. Conclusions

With a recent increase in the life expectancy of patients with CF has come an increased risk for GI cancers, with the US Cystic Fibrosis Foundation now describing CF as a new inherited CRC syndrome. CFTR (ABCC7), a member of the ABC super family, has been shown to act as a tumor suppressor in all the GI cancer types in both patients with CF and in sporadic GI cancers as it is one of the most differentially expressed genes in pancreatic and colon cancer [152]. Notably, CFTR also acts as a tumor suppressor in several major non-GI tract cancers. Loss of CFTR activity due to either inactivating mutations or via epigenetic silencing has been associated with both cancer initiation and progression, and specifically linked to a wide range of oncogenic phenotypes. Given its role as a tumor suppressor, strategies which could upregulate or restore CFTR activity in tumor cells could prove to be immensely beneficial in the clinic. Therefore, new CFTR modulator drugs that have shown great promise in restoring CF-related pulmonary and pancreatic deficiencies in patients with CF may be repurposed for use in the treatment of CFTR-deficient GI cancers in both patients with CF and in patients without CF. These CFTR modulator drugs may also act as chemopreventative agents, as the majority of patients with CF are currently on one modulator drug regimen or another. Overall, these CFTR modulator drugs can represent a new class of personalized cancer medicine.

## Figures and Tables

**Table 1:** The role of CFTR in Esophageal Cancer.

Cancer Type	CFTR-Related Phenotypes
Esophageal Cancer	Expression of CFTR was downregulated in ESCC tissues and was part of an autophagy-related gene prognostic risk signature in esophageal cancer patients [16]. KEGG and STRING bioinformatics analyses identified CFTR as one of the top ten gene hub nodes that are dysregulated in esophageal cancer [19]. CFTR expression was lower in tumor tissues as well as esophageal cancer cell lines. CFTR suppressed the expression of NF-κB-p65 and tumor growth in esophageal cancer cells. KD of CFTR in ESCC cells increased NF-κB pathway signaling and promoted invasive growth in vitro and cancer cell growth in vivo in a mouse xenograft model [14]. CFTR overexpression in ESCC cells suppressed cell proliferation, migration and invasiveness while promoting apoptosis. IHC analysis of the invasive front of primary ESCC tumors showed an inverse relationship between CFTR expression and post-operative survival. Microarray gene expression profiling found that the p38 MAPK pathway was regulated by CFTR which governed ESCC progression [15]. Epidemiological studies indicate that patients with CF are at a higher risk of esophageal cancer and gastroesophageal adenocarcinoma [5,6]. Adults with CF have a 3-fold high risk of developing Barrett’s esophagus which is a precursor for esophageal cancer [17]. GWAS studies reported that the CFTR locus was a risk factor for both Barrett’s esophagus and EAC [18].

**Table 2:** The role of CFTR in Pancreatic, Hepatic and Gastric cancers.

Cancer Type	CFTR-Related Phenotypes
Pancreatic Cancer	CFTR mutations are associated with the development of chronic pancreatitis and pancreatic cancer [21,22]. Epidemiological studies indicate that CF patients are at a higher risk of pancreatic cancer [5,6]. CFTR regulates the expression of MUC4, a glycoprotein associated with tumor progression [25]. Germline heterozygous carriers of CFTR mutations have a high risk of pancreatic cancer, including familial pancreatic cancer [21,22,24].
Hepatic Cancer	Patients with CF have been found to develop hepatocellular carcinoma even in the absence of prior symptoms of liver disease [27,28]. CFTR expression was downregulated by promoter hypermethylation in HCC [3,29]. Epidemiological studies indicate that patients with CF are at a higher risk of biliary tract cancers [5,6]. CFTR prevents biliary tract inflammation and cancer in cholangiocytes via regulating Src tyrosine kinase activity, preventing phosphorylation of TLR4 and suppressing NF-κB pathway activation [30,31].
Gastric Cancer	Germline heterozygous carriers of CFTR F508del have an increased risk of developing gastric cancers [32,33]. CFTR serum levels correlate with the tumor biomarker, CA199 in gastric cancer [33].

**Table 3:** Role of CFTR in Intestinal Cancer.

Cancer Type	CFTR-Related Phenotypes
Intestinal Cancer	A 20-year epidemiological study found that patients with CF are at a 6-fold higher risk for CRC [5,6], a finding confirmed in a separate study of 99,000 adult CF patients [7]. Patients with CF are also at a higher risk of cancers in the small intestine, in particular, the duodenum. Endoscopic screening studies reported a high incidence of early aggressive adenomas in patients with CF patients by age 40, with some having advanced to adenocarcinomas [34,35]. Germline heterozygous carriers of CFTR mutations are at a higher risk of developing CRC [38,39]. Germline heterozygous carriers of specific CFTR polymorphic variants are also at a higher risk for CRC, including in young adults [41] and in patients with familial serrated polyposis syndrome [42]. CFTR also acts as a tumor suppressor and is downregulated in non-CF, non-germline, sporadic human CRC, with CFTR expression linked to DFS and better prognosis [37,42,45]. Expression of CFTR inhibited the proliferation, invasion and migration of CRC cells [43]. CFTR’s role as a tumor suppressor has been confirmed in CF mouse models, either in combination with *Apc^Min^* mutations or in *Apc* wildtype mice [37]. CFTR was reported to interact with AF6/afadin in regulating colon cancer metastasis [43]. Loss of CFTR in the intestine is associated with increased Wnt/β-catenin signaling [46].
